# HIV-related stigma and associated factors: a systematic review and meta-analysis

**DOI:** 10.3389/fpubh.2024.1356430

**Published:** 2024-07-23

**Authors:** Zelalem G. Dessie, Temesgen Zewotir

**Affiliations:** ^1^College of Science, Bahir Dar University, Bahir Dar, Ethiopia; ^2^School of Mathematics, Statistics and Computer Science, University of KwaZulu-Natal, Durban, South Africa

**Keywords:** Begg's test, effect size, Egger's test, heterogeneity, funnel plot, publication bias, sensitivity analysis

## Abstract

**Background:**

It has been recognized that HIV-related stigma hinders efforts in testing, treatment, and prevention. In this systematic review, we aimed to summarize available findings on the association between HIV-related stigma and age, social support, educational status, depression, employment status, wealth index, gender, residence, knowledge about HIV, marital status, duration since diagnosis, and disclosure status using a large number of studies.

**Methods:**

Electronic databases including Scopus, Medline/PubMed, Web of Sciences (WOS), Cochrane Library, Google Scholar, and Open Research Dataset Challenge were systematically searched until 15 April 2023. We included all kinds of HIV-stigma studies, regardless of language, publishing date, or geographic location. The inclusion criteria were met by 40 studies, with a total of 171,627 patients. A mixed-effect model was used to pool estimates and evaluate publication bias, as well as to conduct sensitivity analysis.

**Results:**

Factors such as older age, social support, greater education, higher socioeconomic status, good knowledge of HIV, and longer years of living with HIV significantly lowered the likelihood of HIV-related stigma. Contrarily, factors such as depression, residing in rural areas, female respondents, and non-disclosure of HIV status were significantly associated with a high risk of HIV-related stigma.

**Conclusion:**

To combat systemic HIV-associated stigma, it is crucial to develop wholesome and comprehensive social methods by raising community-level HIV awareness. In addition to activism, local economic development is also crucial for creating thriving communities with a strong social fabric.

## Introduction

HIV-related stigma and discrimination pose significant obstacles to HIV responses. Progress has thus far been stalled by fragmented efforts to fight HIV-related stigma and prejudice (1). It is important to analyze the various existing measures to derive lessons that can guide future interventions and strengthen the evidence base on HIV-related stigma.

HIV-related stigma is defined by stigmatizing actions such as treating people differently, verbally abusing them, undervaluing them, and rejecting them in social settings (2, 3). According to the Joint United Nations Programme on HIV/AIDS (UNAIDS), HIV-related stigma is defined as negative attitudes, feelings, and beliefs toward people living with HIV, groups associated with people living with HIV (PLWH), and other important populations at a higher risk of contracting HIV (1). There are three wars through which people who live with HIV may feel stigmatized: Internalized stigma, anticipated stigma, and enacted stigma (4). Internalized stigma is the acceptance of unfavorable thoughts, opinions, and sentiments about oneself in relation to one's HIV-positive status (5). Anticipated stigma is the awareness of unfavorable social attitudes regarding HIV and the expectation that an HIV-positive individual would eventually face prejudice and discrimination (6). Enacted stigma describes the prejudice that HIV-positive individuals encounter, and it may take the form of violence and marginalization (7).

Experiences of stigmatization have been linked to poorer quality of life, poorer mental health and wellbeing, and reduced access to care for people with HIV (8). Numerous studies have emphasized the detrimental effects of HIV-related stigma on those who are living with the virus. For instance, in recent research on HIV-related stigma, stigma was found to be associated with poorer mental health outcomes, including anxiety (6, 9), depression (10, 11), emotional distress (12), and life satisfaction (13, 14). Research conducted by Turan et al. (15) demonstrated that HIV-related stigma within communities can lead individuals living with HIV to internalize stigma and expect stigmatizing encounters, which can subsequently lead to negative health and psychosocial consequences. Furthermore, a meta-analysis by Rueda et al. (6) found high levels of stigma to be linked to risky sexual behavior, depression, lower medication adherence, reduced use of health and social services, anxiety, negative self-image, poorer quality of life, mental distress, and reduced social support from friends, family, and health services. These findings imply that stigma associated with HIV can have a negative effect on people living with HIV in terms of a variety of health-related outcomes.

Although several studies (8, 16–21) have been carried out to identify potential risk factors of HIV-related stigma, there remains a scarcity of research demonstrating the common causes of HIV-related stigma. Numerous studies have investigated various factors associated with HIV-related stigma; however, they have often been confined to specific geographic areas, cases, or characteristics. Therefore, the objective of this study was to evaluate a wide range of HIV-related stigma risk factors by summarizing findings from over 40 scientific research articles.

## Methods

### Study protocol

To investigate the relationship between high levels of HIV-related stigma and age, social support, educational status, depression, employment status, wealth index, gender, residence, knowledge about HIV, marital status, duration since diagnosis, and disclosure status, we followed PRISMA criteria (22) to perform a meta-analysis of articles identified through our reviews.

### Search strategy

A comprehensive search was conducted until 15 April 2023, across the following electronic databases: EMBASE, Scopus, Medline/PubMed, Web of Sciences (WOS), Google Scholar, Cochrane Library, and Open Research Dataset. The search terms used in the strategy were “HIV-related stigma” and/or “internalized stigma,” “anticipated stigma,” and “enacted stigma.” In addition, the search was limited to studies that considered sociodemographic traits, risk factors, and clinical aspects as possible indicators of high levels of stigma associated with HIV. No restrictions were applied regarding the timing and language of the publications. We downloaded the literature results into EndNote X9 to speed up the screening process and save time.

### Eligibility criteria

**Figure 1:**
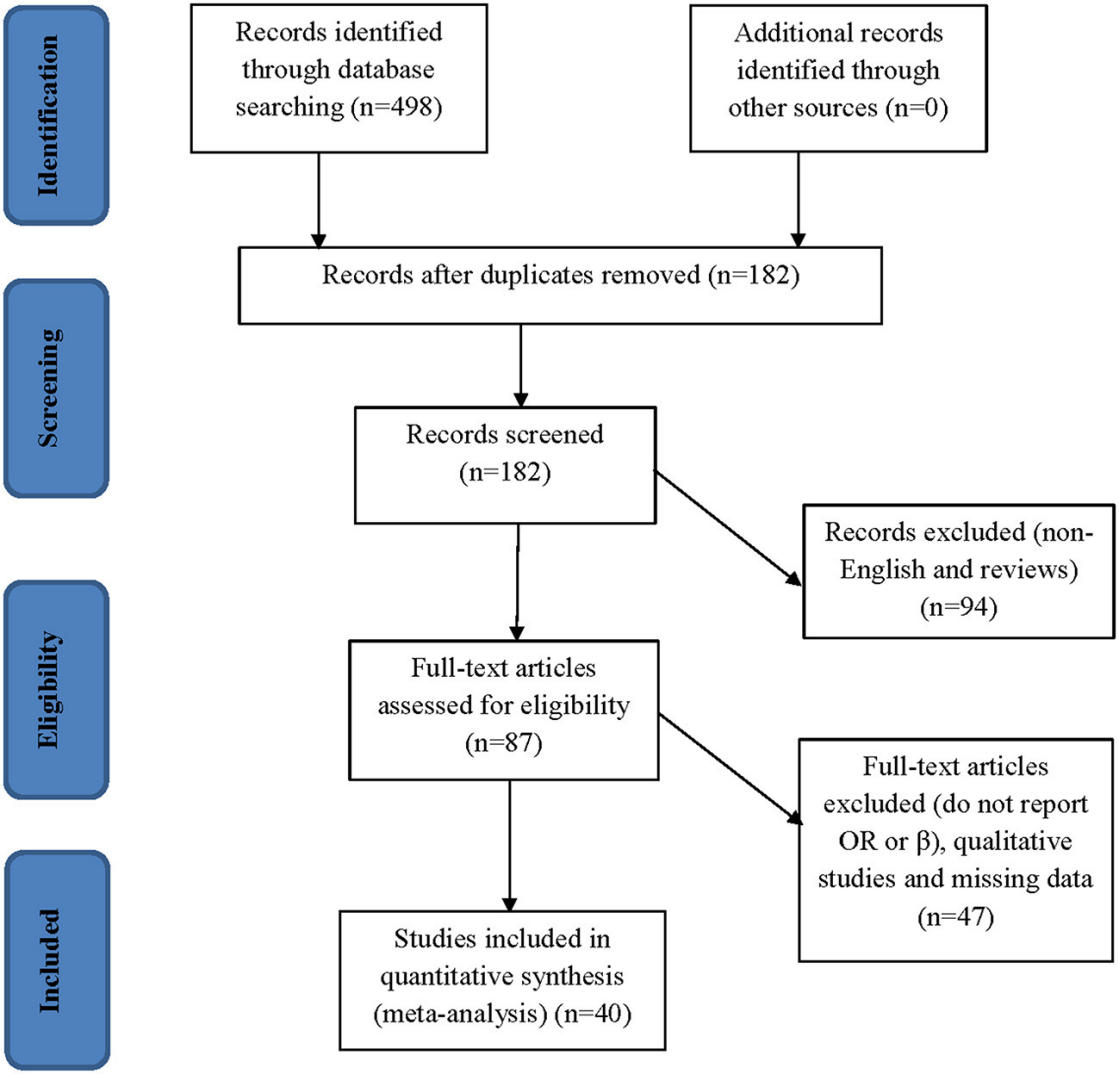
Flow chart of the study search and screening process.

The initial search results were evaluated for relevancy using titles and abstracts after removing duplicates. The eligibility requirements were examined in all the studies (Figure 1). The analysis did not include studies without a full text and/or abstract, expert opinion pieces, novels, theses, editorials, or review articles. It also did not include correspondence letters. In addition, we only utilized studies from the eligible literature if they provided odds ratios (ORs) or estimated coefficients (β) together with 95% confidence intervals (CI) for the correlation between risk factors, epidemiological factors, or demographic variables and HIV-related stigma.

### Assessment for study quality and data extraction

Both authors separately evaluated the downloaded EndNoteX9 search outputs' suitability for inclusion. Any disagreements between the authors were resolved through dialogue and consensus. Both extracted information about the sample size, outcome, publication year, author's name, countries, study design, variables (e.g., age, social support, educational status, depression, employment status, wealth index, gender, residence, knowledge about HIV, marital status, duration since diagnosis, and disclosure status), and odds ratios (ORs) or estimated coefficients (β). The authors independently assessed the quality of the methodological approaches of the studies by using the Newcastle–Ottawa method (23). In this method, the comparability of study groups, the evaluation of results, and patient selection were used as three key components to gauge the quality of the studies. Each of the seven domains in the Newcastle–Ottawa method was given a score between 3 and 0 (from low to high bias), and the average score was then calculated.

### Instruments

The eligible studies used various measures to assess HV/AIDS stigma, and the most common were the Berger HIV Stigma Scale (24), the HIV Stigma Measure (25, 26), and the Internalized HIV Stigma instrument (27). When studies reported multiple forms of HIV-related stigma, such as internalized stigma, personalized stigma, self-stigma, enacted stigma, and/or aggregated stigma, the finding that was most strongly associated with the outcome was chosen. The tools in the Berger HIV Stigma Scale consist of four subscales: (1) negative self-image, (2) disclosure concerns, (3) public attitudes, and (4) personalized stigma. Each question can be rated based on a 4-point Likert scale (strongly disagree, disagree, agree, and strongly agree). The overall stigma score is dichotomized as “high level of stigma” if the participants' score is greater than or equal to the mean score scales, or otherwise “low level of stigma (24).” Moreover, the adapted 10-item HIV Stigma Scale by Wright et al. (26) gauges anticipated stigma, personalized stigma, and negative self-image while also generating an overall stigma assessment. Each item employs a 5-point Likert scale, spanning from 1 (“strongly disagree”) to 5 (“strongly agree”). An overall stigma score from 0 to 100 is computed by adjusting each item's values. Based on the mean, both an overall stigma measure and sub-dimensional measures are categorized, which results in a binary assessment of higher or lower stigma levels.

### Statistical analysis

The effect size statistic was defined as the association between HIV-related stigma and covariates. We examined the link between HIV-related stigma and risk factors from peer-reviewed published research using odds ratios (ORs) or estimated coefficients (β) (with 95%CI). We calculated a mixed-effect model, taking into account the anticipated between-study heterogeneity. We calculated Cochran's Q test and the I^2^ statistic measure to evaluate the heterogeneity. The Cochran's Q test was employed to ascertain whether there was heterogeneity in effect sizes; a significant Q value indicated heterogeneity as opposed to homogeneity. The I^2^ statistic was employed to determine the percentage of the total variance that could be attributable to study heterogeneity (28). In addition, mild, moderate, and severe conditions were assigned to I^2^ levels between 0% and 39%, 40% and 59%, and 60% and 90%, respectively (28). Funnel plots using Egger's weighted regression test were employed to assess publication bias (29). A *p*-value of 0.05 was considered statistically significant. For each analysis, STATA version 17 and R-4.3.0 statistical tools were used to calculate the pooled estimate and analyze publication bias.

## Results

### Search results

Electronic databases [such as Scopus database, Medline/PubMed, EMBASE, Web of Sciences (WOS), Cochrane Library, and Google Scholar] were used to retrieve a total of 498 titles and abstracts. Duplicate records were eliminated, leaving 182 records that might be relevant. After additional screening, 94 full-text articles were used for the eligibility assessment. These were evaluated under the exclusion criteria, and 87 studies were selected for inclusion. From these 87 studies, 18 qualitative studies and 14 studies with missing data were excluded. A total of 15 studies did not include ORs or β and were thus excluded. Consequently, we only included 40 studies that met all the eligibility requirements (see Figure 1).

### Geographical distribution and demographic characteristics

A thorough description of all the chosen studies (3–8, 10, 12, 14, 16, 30–45) is provided in Table 1. Most of the studies were released between 2018 and 2022. All the included studies reported a total number of 171,627 patients. Five of them were carried out in the United States, seven in China, four in South Africa, nine in Ethiopia, four in Uganda, two each in Canada and Thailand, one each in India, Kenya, Morocco, Sub-Saharan Africa, Cameroon, and Tanzania, and one in a mixed region. Between 123 and 56,367 patients were included in the sample. In the studies, the participants' age ranged from 42.8 to 68 years (Table 1).

**Table 1:** Characteristics of studies included in the systematic review and meta-analysis.

**References**	**Country**	**Sample size**	**Type of stigma**	**Stigma measure**	**Mean (±SD) of HIV stigma/Prevalence of HIV stigma**	**Mean (±SD) /Median [IQR] of age**	**Associated Factors**
Takada et al. (42)	Uganda	422	Internalized stigma	Internalized HIV Stigma instrument	1.3(1.9)^a^	35(34)^a^	Social support: β = −0.13 (−0.25, −0.005) Higher education: β = −0.15(−0.26, −0.04) Probable depression: β = 0.46 (0.26, 0.67) Household asset: β = −0.05 (−0.07, −0.02)
Wedajo (46)	Ethiopia	714	Perceived stigma	HIV Stigma Measure	11.2(4.1)^a^	37 [30–45]	Social support: β = −0.54 (– 0.66, – 0.41)
Arinaitwe et al. (32)	Uganda	252	Overall stigma	Berger HIV Stigma Scale	70.08 (19.34)^a^		Social support: aOR = 0.99 (0.98–0.99) Old age: aOR = 0.89 (0.83–0.96) Food insecurity: aOR = 1.07(1.01–1.15)
Li (36)	Thailand	408	Perceived stigma	Herek and Capitanio	21.5(4.9)^a^		Social support: −0.09 Income: −0.09
Tsai (47)	sub–Saharan Africa	4313	Internalized stigma	Internalized HIV Stigma instrument	19.8%	33.5 [33.2–33.8]	Higher education: aOR = 0.36 (0.18–0.71) Occupation: Professional: aOR = 0.79 (0.64–0.97) Household asset wealth: aOR = 0.41 (0.32–0.53)
Nikus Fido et al. (40)	Ethiopia	318	Overall stigma	Berger HIV Stigma Scale	50.5(16.4)^a^		Lower education: β = 30.03 (22.3–37.7) Occupation: Others: β = 17.63 (5.6–29.7) Social support: β = −7.00(−11.4, −2.6)
Ncitakalo et al. (48)	South Africa		Overall stigma	HIV Stigma Measure	37.9%		Higher education: aOR = 0.60 (0.41–0.88) Household asset wealth: aOR = 0.69 (0.50–0.96) Residence: Rural: aOR = 2.07 (1.25–3.41) Self–perceived risk of HIV: Yes: aOR = 1.36 (1.03–1.78) Correct knowledge about HIV: Yes: aOR = 0.54 (0.37–0.80)
Li et al. (49)	China	4050	Overall stigma	HIV Stigma Measure	37.0%		Old age: aOR = 1.26 (1.01–1.57) Higher education: aOR = 0.62 (0.53–0.73) Correct knowledge about HIV: Yes: aOR = 0.94 (0.93–0.95)
Yin et al. (45)	China	1248	Overall stigma	HIV Stigma Measure	50.7(8.3)	30.2(7.2)^a^	Correct knowledge about HIV: Yes: β = −0.66 (SE = 0.08)
Rayanakorn et al. (19)	Thailand	161	Overall stigma	Berger HIV Stigma Scale	28.31(5.43)^a^		Old age: β = −1.81 (−3.34, −0.28) Household asset wealth: β = 4.89 (2.28–7.52) Occupation: Professional: β = 6.93 (2.39–11.47)
Moussa et al. (39)	Morocco	626	Internalized stigma	Berger HIV Stigma Scale	88.2%	36 [28–43]	No formal education: aOR = 1.38 (1.16,1.65) Occupation: full–time: aOR = 0.84 (0.73,0.96) Reactions of other adult family members: discriminatory: aOR = 1.28 (1.11,1.49)
Xu et al. (44)	China	277	Internalized stigma	Internalized HIV Stigma instrument	32.39(7.16)^a^	30 [26–38]	Old age: β = 0.08 (0.01–0.16) Social support: β = −0.19(−0.29, −0.10) Increase Stereotype: β = 0.51(0.38–0.65)
Peltzer and Pengpid (20)	South Africa	10473	Overall stigma	HIV Stigma Measure	30.5%		Old age: aOR = 0.55 (0.39, 0.68) Gender: Women: aOR = 1.32 (1.16, 1.50) Marital status: Single: aOR = 1.51 (1.30, 1.75) Lower number of years of living with HIV: aOR = 1.41 (1.19, 1.66) Household wealth index: aOR = 0.72 (0.58, 0.89) Higher education: aOR = 0.75 (0.62, 0.91) Residence: Urban: aOR = 0.73 (0.63, 0.84) Correct knowledge about HIV: Yes: aOR = 0.77 (0.67, 0.89)
Adhikari et al. (30)	India	444	Overall stigma	Berger HIV Stigma Scale	32.7%		Old age: aOR = 1.54 (1.07,2.28) Household wealth index: aOR = 0.75 (0.57, 0.97) Comorbidities: aOR = 8.49 (3.54, 20.38)
Li et al. (50)	China	522	Perceived stigma	Herek and Capitanio	28.95(6.03)^a^	48.38^a^	Gender: Women: β = 1.62 (SE = 0.67)
Hargreaves et al. (51)	South Africa and Zambia	3859	Internalized stigma	Internalized HIV Stigma instrument	22.5%		Old age: aOR = 1.58 (1.15, 2.17) Gender: Women: aOR = 1.22 (1.04, 1.43) Many sexual partners in life: aOR = 2.73 (1.19–6.26) Household wealth index: aOR = 1.12 (0.84–1.51)
Minja et al. (38)	Tanzania	742	Overall stigma	Berger HIV Stigma Scale	2.1(0.7)^a^	29.6 [18–24]	Disclosure: β = −1.21 (−1.92, −0.5) Social support: β = −0.35 (−0.58, −0.12) Depression: β = 0.09 (0.06–0.12)
Antabe et al. (31)	Malawi	24,036	Overall stigma	Berger HIV Stigma Scale	19%		Old age: aOR = 0.98 (0.97, 0.99) No formal education: aOR = 2.81 (2.50,3.15) Correct knowledge about HIV: No: aOR = 1.64 (1.52;1.76) Household wealth index: poorest aOR = 3.21 (2.83, 3.64) Occupation: unemployed: aOR = 1.13 (1.05, 1.21) Residence: Rural: aOR = 2.08 (1.82, 2.37) Marital status: Single: aOR = 1.23 (1.08, 1.39)
Li et al. (37)	China	239	Overall stigma	Berger HIV Stigma Scale	48.66(6.29)^a^	51.76(6.96)^a^	Old age: β = −0.57 (−0.78,−0.35) Years since HIV diagnosis: β = −0.13(−0.26, −0.01)
Nyasulu et al. (52)	South Africa	1146	Overall stigma	Berger HIV Stigma Scale	51.0%		Occupation: employed: aOR = 0.78 (0.71–0.87) knowledge about HIV: aOR = 0.85 (0.75–0.97) Marital status: Married: aOR = 1.14 ( 1.02–1.28)
Feyasa et al. (34)	Ethiopia	28371	Overall stigma	Berger HIV Stigma Scale	35.65%		Residence: Rural: aOR = 1.82 (1.46, 2.27) Higher education: aOR = 0.43 (0.32, 0.56) Marital status: Married: aOR = 1.38 (1.19, 1.61) Old age: aOR = 0.81 (0.73, 0.91)
Tao et al. (53)	China	367	Overall stigma	HIV Stigma Scale	17(0.25)^a^	28 [25–32]	Depression: aOR = 1.09 (1.07–1.12)
Yator et al. (54)	Kenya	123	Internalized stigma	HIV Stigma Scale	0.75(0.40)^a^	31 [19–48]	Depression: β = 0.64 (0.38–0.89) Old age: β = −0.04 (−0.06, −0.01) Marital status: Married: β = 0.27 (0.01, 0.54) High Income: β = −0.25 (−0.5, −0.02) Social support: β = −0.31 (−0.55, −0.06)
Alemu et al. (16)	Ethiopia	638	Perceived stigma	Berger HIV Stigma Scale	57.8%		lower education: aOR = 3.36 (2.07, 5.42) Disclosure: aOR = 1.66 (1.12, 2.45) Social support: No: aOR = 2.05(1.19, 2.43)
Turi et al. (43)	Ethiopia	418	Perceived stigma	Berger HIV Stigma Scale	48.6%		Gender: Women: aOR = 2.10(1.15, 3.82) Lower duration of treatment: aOR = 2.63 (1.09, 6.34) Depression: aOR = 1.85(1.08, 3.19) Social support: No: aOR = 2.22(1.09, 4.54) Disclosure: No: aOR = 2.00 (1.11, 3.89)
Ajong et al. (55)	Cameroon	308	Overall stigma	Berger HIV Stigma Scale	49.8%	40.1(10.2)^a^	Higher education: aOR = 0.70(0.44–0.91) Lower number of years since HIV diagnosis: aOR = 1.74 (1.01–3.00)
Chekole and Tarekegn (56)	Ethiopia	403	Perceived stigma	Berger HIV Stigma Scale	42.7%		Gender: Women: aOR = 2.36 (1.28–4.33) Older age: aOR = 1.11 (1.26–4.65) Lower education: aOR = 7.50 (3.45–9.74)
Nabunya et al. (57)	Uganda	702	Internalized stigma	Berger HIV Stigma Scale	12.19(3.79)^a^		Social support: Yes: β = −0.05(−0.09, −0.01) Disclosure: No: β = 0.20 (0.03, 0.38)
Abubakari et al. (58)	USA	1437	Perceived stigma	Berger HIV Stigma Scale	35%		HIV Knowledge: Yes: β = −0.24(SE = 0.06) Bringing up the topic of HIV: β = −0.13(0.04) Older age: β = 0.04 (SE = 0.01)
Adane et al. (17)	Ethiopia	422	Perceived stigma	Berger HIV Stigma Scale	41.93%		Gender: Women: aOR = 2.08 (1.26–3.46) Residence: Rural: aOR = 1.801 (1.10–2.94) Disclose HIV: No: aOR = 2.39 (1.19–4.7)
Williams et al. (21)	USA	603	Overall stigma	HIV Stigma Scale	53.7%	49(0.64)^a^	Gender: male: aOR = 0.90 (0.89–0.92) Older age: aOR = 0.73 (0.72–0.74) Higher education: aOR = 0.97 (0.97–0.98) Depression: aOR = 1.21 (1.19–1.22)
Algarin et al. (59)	USA	932	Enacted stigma	Herek HIV related stigma measure	53.1%		Depression: aOR = 1.61 [1.19, 2.18]
Dahlui et al. (33)	Nigeria	56 307	Perceived stigma	Berger HIV Stigma Scale	50%		Gender: male: aOR = 1.76(1.68–1.84) Younger age: aOR = 1.20(1.14–1.26) Lower education: aOR = 1.75(1.58–1.94) Household wealth index: poorest aOR = 1.92 (1.80–2.05)
Williams et al. (60)	USA	603	Overall stigma	HIV Stigma Scale	49%		Gender: male: aOR = 0.77 (0.50–1.19) Older age: aOR = 0.28 (0.09–0.82) Depression: aOR = 2.87 (1.38–5.98)
Deering et al. (61)	Canada	215	Overall stigma	HIV Stigma Scale	30.7%	46 [39–53]	Duration since HIV diagnosis: aOR = 0.97(0.94–0.99) Non–disclosure of HIV: aOR = 2.48(1.57–3.94)
Small et al. (62)	Uganda	8058	Overall stigma	Berger HIV Stigma Scale	41.3%		Poor Wealth index: aOR = 1.36(1.03–1.80)
Yang et al. (63)	China	318	Overall stigma	Berger HIV Stigma Scale	105.13 (21.58)^a^	37 [33–45]	Time since HIV: β = −0.06(−0.11,−0.01) Disclosure: β = 7.65(2.86, 12.42) Social support: β = −1.12(−1.57, −0.68)
Duko et al. (64)	Ethiopia	401	Overall stigma	Berger HIV Stigma Scale	43.9%	38 (10.23)^a^	Depression: aOR = 2.83 (1.78, 4.48)
Spence et al. (41)	USA	236	Overall stigma	Berger HIV Stigma Scale	67.1(8.2)^a^	42.3(12.3)^a^	Gender: Women: β = 4.97 (0.61, 9.32) Knowledge about HIV: β = 0.79 (−1.16,2.76)
Gurmu and Etana (35)	Ethiopia	16,515	Overall stigma	Berger HIV Stigma Scale	72.1%		Older age: aOR = 0.71 (SE = 0.10) Higher education: aOR = 0.41 (SE = 0.12) Wealth Index: rich: aOR = 0.83(SE = 0.09) Knowledge of HIV: Yes: aOR = 2.89 (0.18)

aOR, adjusted odds ratio; SD, Standard deviation; IQR, interquartile range. Stigma: ^a^Reported as mean (± SD) of the stigma level. Other studies were reported as prevalence of HIV-related stigma. Age: ^a^Reported as mean (± SD). Other studies were reported as median (IQR).

### Pooled prevalence of high HIV-related stigma level

**Figure 2:**
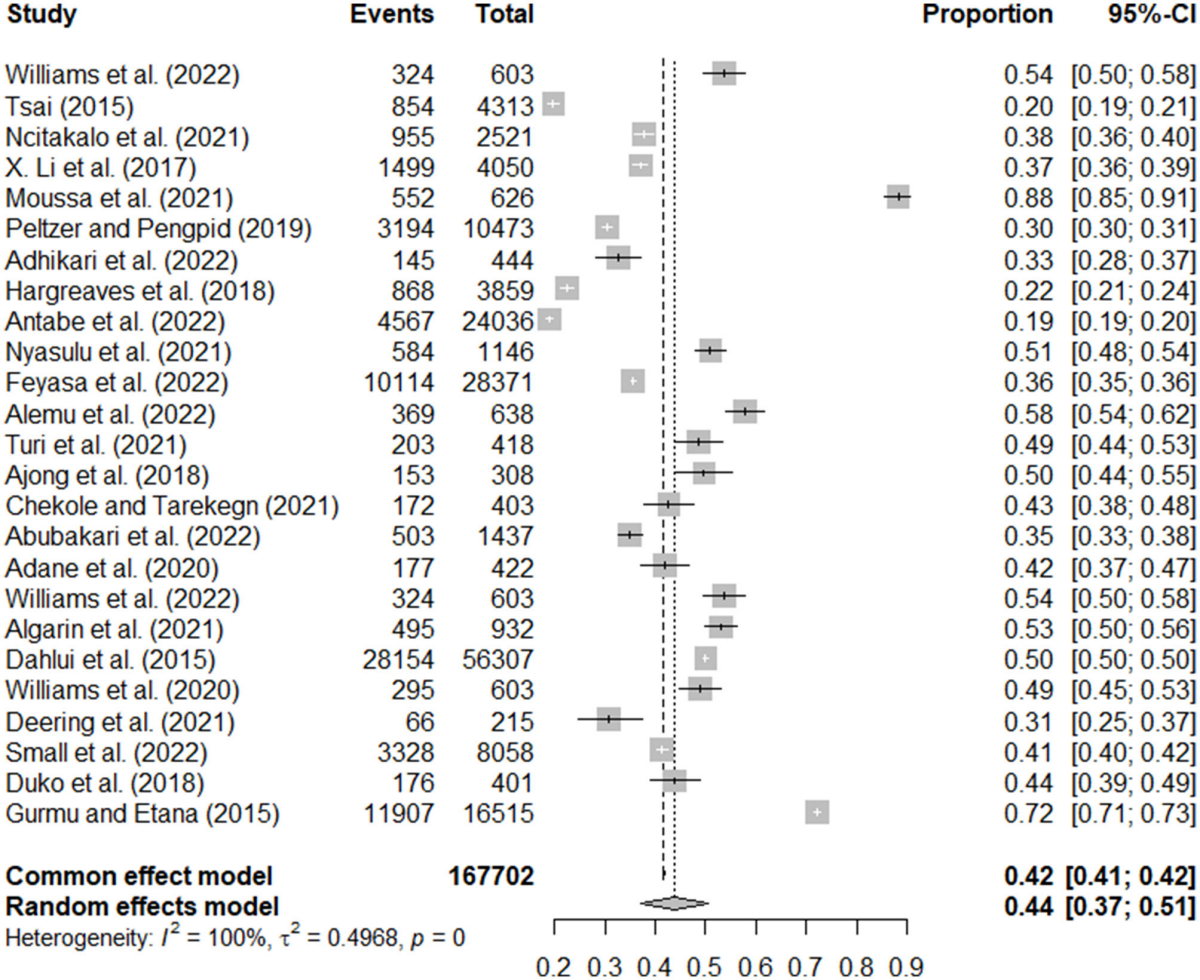
The pooled prevalence of HIV-related stigma.

The results from the mixed effect meta-analysis model are shown in Figure 2. We deduced from this plot that, among the studies considered, the prevalence of HIV stigma ranged from a minimum level of 19% (95%CI: 18.9%−20.1%) (31) to a maximum level of 88% (95%CI: 85%−91%) (37). Approximately 171,627 patients were enrolled in the study, of which 69, 978 patients reported high stigma levels, yielding a weighted pooled overall high stigma level of 44% (95% CI, 37%−51%) (Figure 2).

### Risk factors for HIV-related stigma

**Figure 3:**
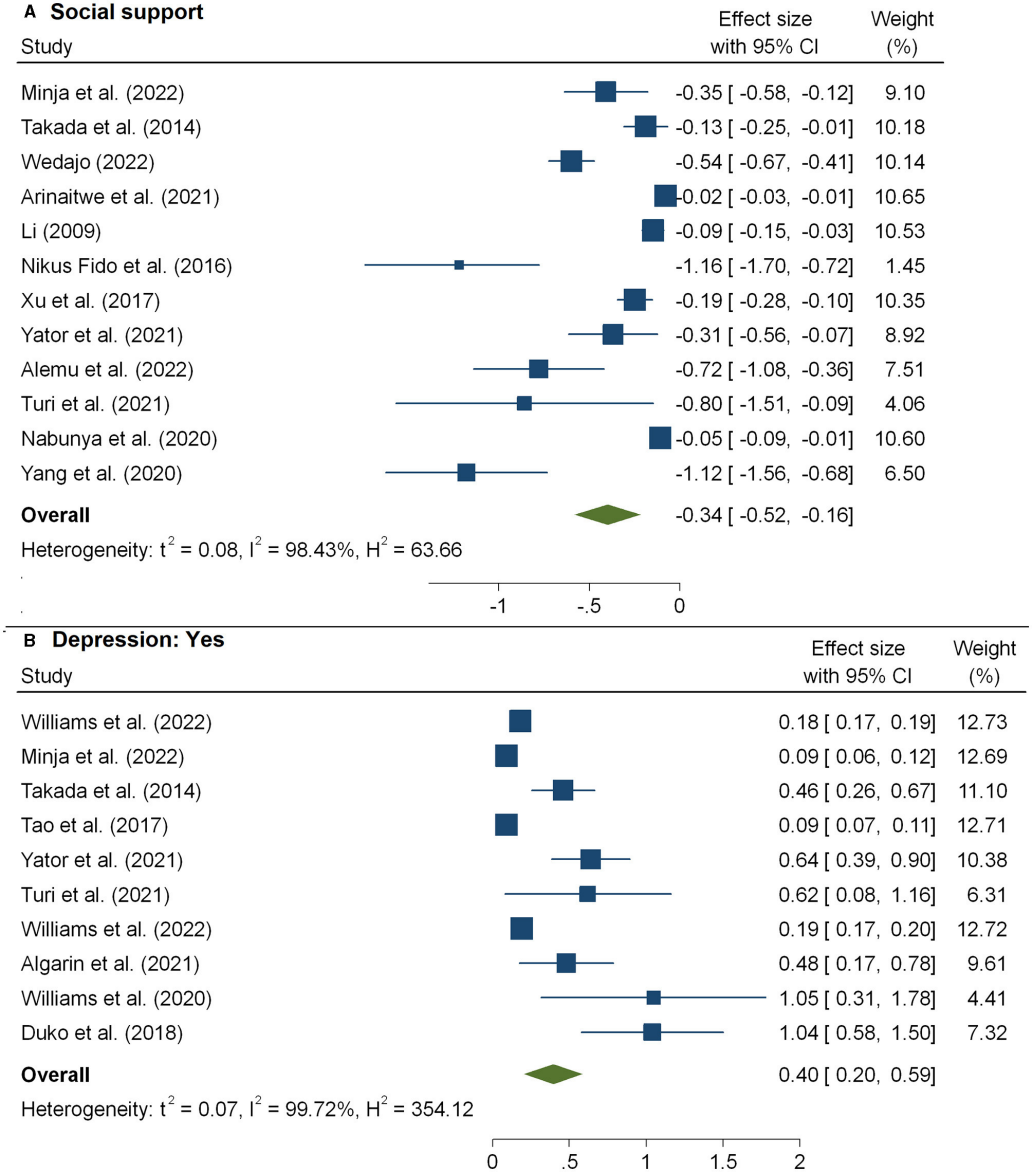
The pooled effect size showing the association between HIV-related stigma and social support **(A)** and depression **(B)**.

**Figure 4:**
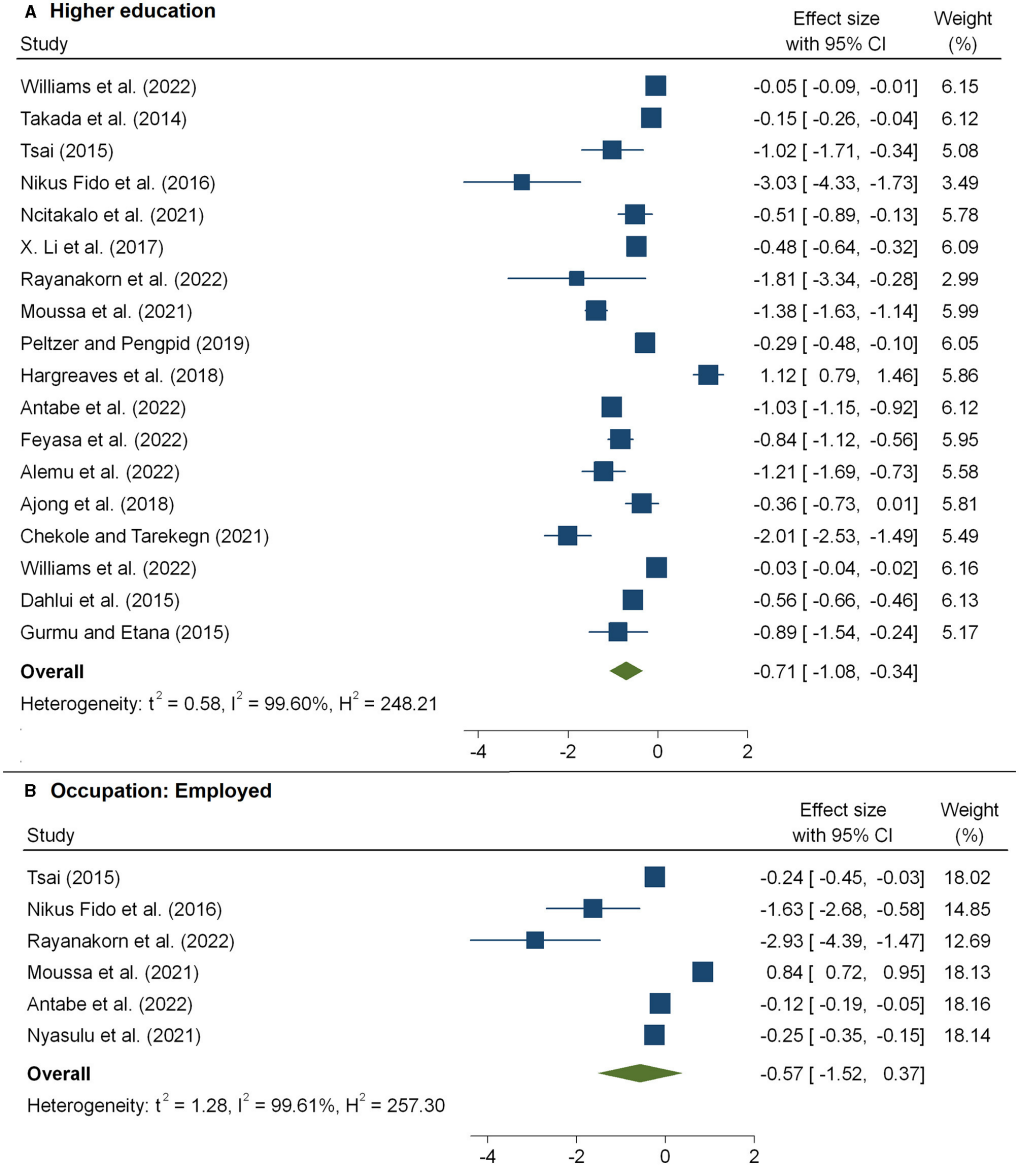
The pooled effect size showing the association between HIV-related stigma and educational level **(A)** and occupational status **(B)**.

**Figure 5:**
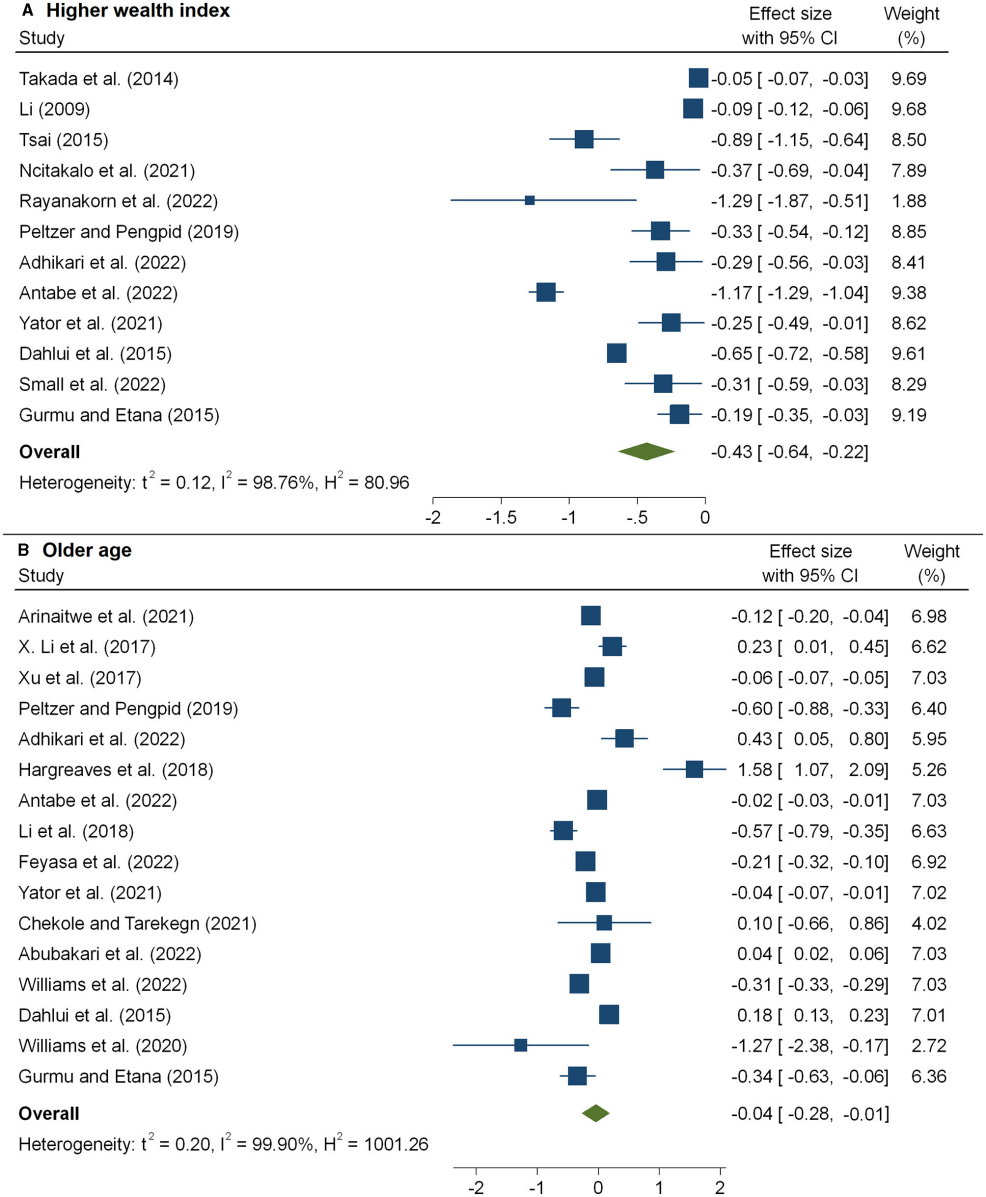
The pooled effect size showing the association between HIV-related stigma and wealth index **(A)** and age **(B)**.

The meta-analysis of 12 studies (16, 32, 36, 38, 40, 42–44, 46, 54, 57, 65) that examined the association between HIV-related stigma and social support among people living with HIV is presented in Figure 3A and Table 2. From the results, we observed that social support reduced the impacts of poor care retention and HIV-related stigma [pooled β: −0.34(95%CI: −0.52, −0.16)]. In Figure 3B, the association between HIV-related stigma and depression is shown to be positive. Depression showed an increased level of stigma, and the pooled estimate was 0.49(95%CI: 0.20, 059). The combined 18 effect sizes from 18 studies (16, 19, 31, 33–35, 39, 40, 42, 47, 48, 51) revealed that the level of HIV-related stigma significantly decreased with an increasing level of education (pooled β: −0.71(95%CI: −1.08, −0.34) (Table 2 and Figure 4A). Furthermore, from socioeconomic factors, it was observed that the level of HIV-related stigma significantly decreased among patients who were richer [pooled β: −0.43(95%CI: −0.64, −0.22)] (Figure 5A) and employed (pooled β: −0.57(95%CI: −1.52, −0.37) (Figure 4B), as compared to patients who were poorer and unemployed.

**Table 2:** Results of the subgroup analysis based on clinical and demographic variables associated with high stigma levels.

**Risk factors**	**Numbers of study**	**Effect size (95% CI)**	**Heterogeneity**		**Egger's test *p*-value^#^**	**Begg's test *p*-value^#^**
			**I** ^2^	***p*** **-value**		
Social support: Yes vs. No	12	−0.34(−0.52, −0.16)	98.43	0.000	0.421	0.621
Depression: Yes vs. No	10	0.49(0.20, 059)	99.72	0.000	0.293	0.272
Higher education	18	−0.71(−1.08, −0.34)	99.60	0.000	0.088	0.090
Employed occupational status	6	−0.57(−1.52, −0.37)	99.61	0.000	0.312	0.101
Higher wealth index	12	−0.43(−0.64, −0.22)	98.76	0.000	0.051	0.049
Older age	16	−0.04(−0.28, −0.01)	99.00	0.000	0.341	0.231
Urban residence	5	−0.33(−0.81, −0.15)	95.94	0.000	0.341	0.231
Good knowledge of HIV	9	−0.36(−0.51, −0.20)	94.51	0.000	0.410	0.388
Marital status	5	0.01(−0.26, 0.29)	94.24	0.000	0.343	0.413
Female	11	0.47(0.11, 0.84)	99.79	0.000	0.123	0.076
Duration since diagnosis	6	−0.17(−0.31, −0.03)	92.14	0.000	0.231	0.221
Disclosure: No vs. Yes	7	0.75(0.41, 1.09)	71.13	0.000	0.432	0.471

The effect size statistic was defined as the association between HIV-related stigma and covariates. (#) H0 there are no small study effects.

**Figure 6:**
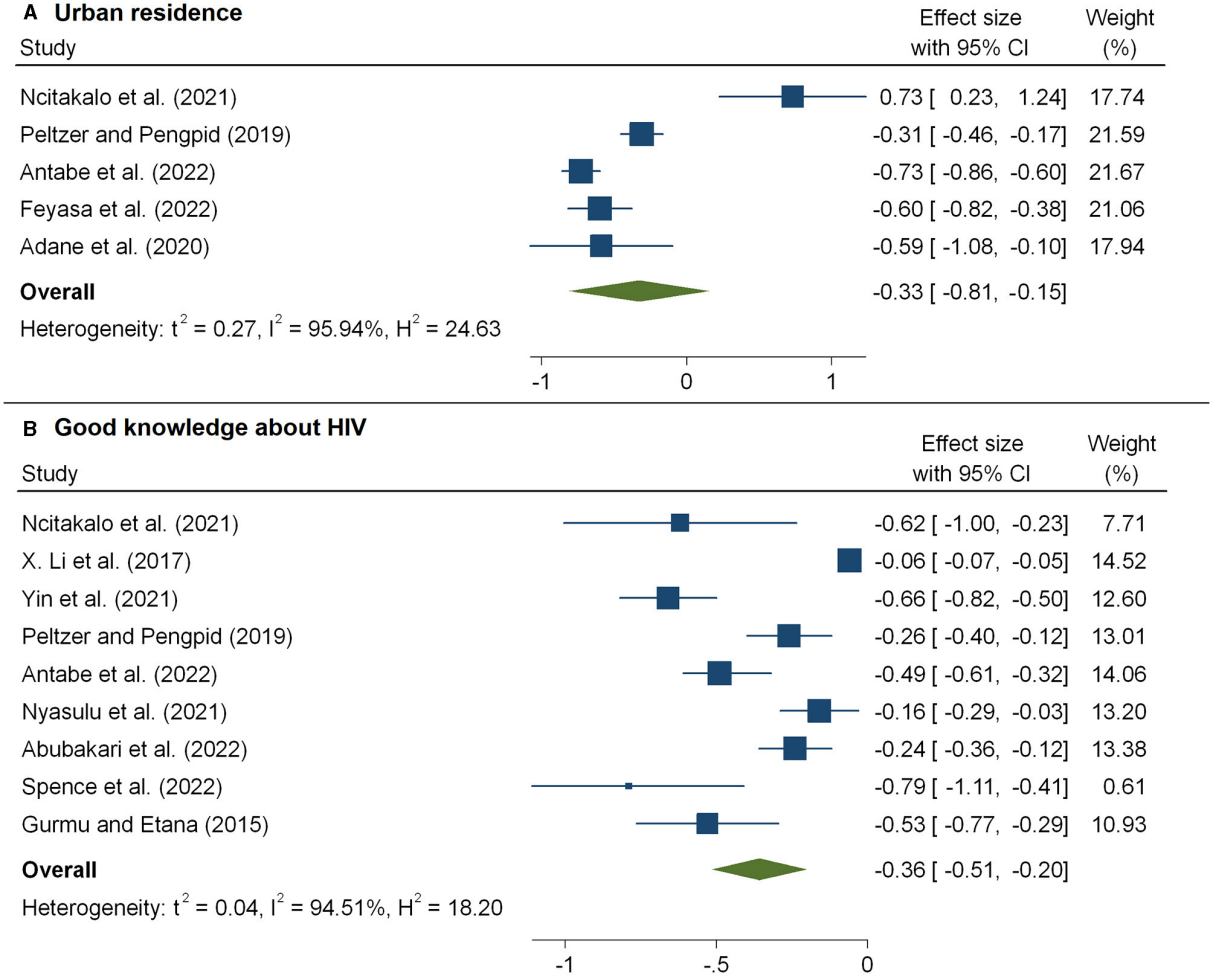
The pooled effect size showing the association between HIV-related stigma and residence **(A)** and knowledge about HIV **(B)**.

The meta-analysis of 16 studies (30–35, 37, 44, 51, 54) that examined the association between the level of HIV-related stigma and age is presented in Figure 5B and Table 2. From the results, we observed that older age was associated with a decreased level of stigma, and the pooled estimate was −0.04 (95%CI: −0.28, −0.01). According to five studies (17, 20, 31, 34, 48), patients living in urban areas faced a considerably decreased level of stigma, and the pooled estimate was −0.33(95%CI: −0.81, −0.15) (Figure 6A and Table 2).

**Figure 7:**
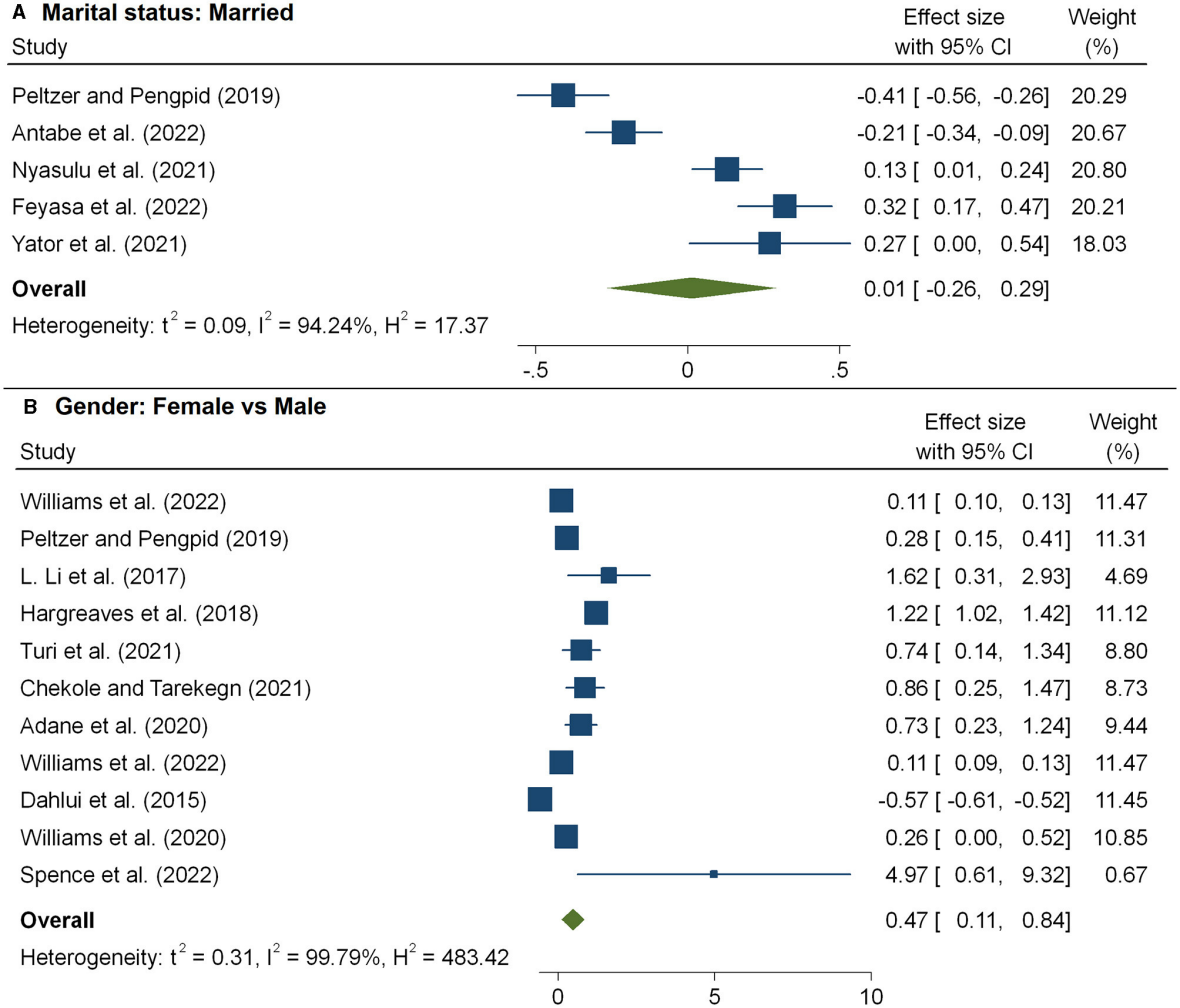
The pooled effect size showing the association between HIV-related stigma and marital status **(A)** and gender **(B)**.

**Figure 8:**
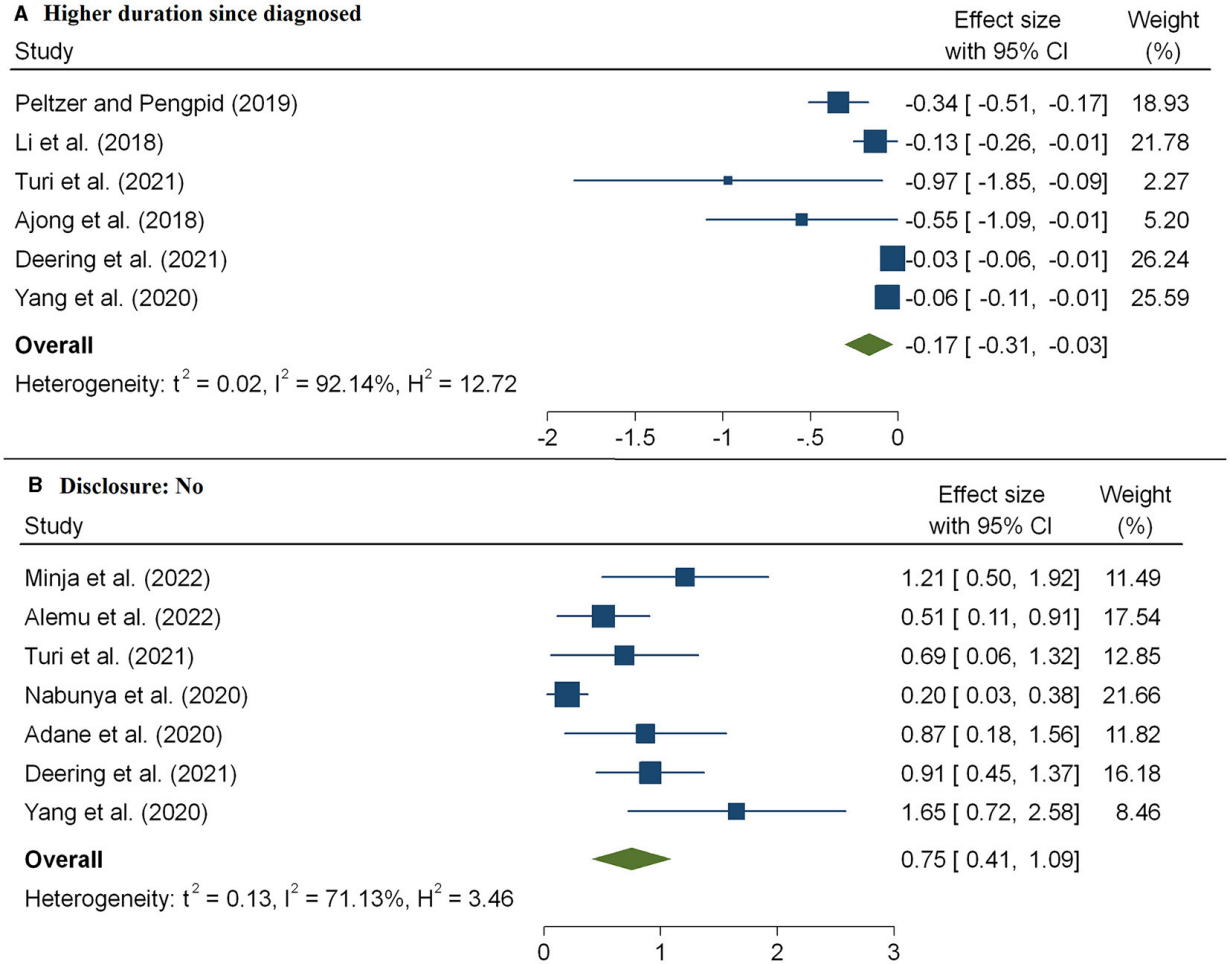
The pooled effect size showing the association between HIV-related stigma and duration since diagnosis **(A)** and disclosure status **(B)**.

The HIV/AIDS knowledge score significantly predicted the level of HIV-related stigma. As shown in Figure 6B, we observed that the level of stigma significantly decreased with an increasing HIV/AIDS knowledge score, and the pooled estimate was −0.36(95%CI: −0.51, −0.20). The combined 11 effect sizes from 11 studies (17, 20, 21, 33, 41, 43, 51) revealed that women were more likely to face a high level of stigma, and the pooled estimate was 0.47(95%CI: 0.11, 0.84) (Figure 7B). However, the association of marital status with HIV-related stigma was not found to be significant (pooled β: 0.01; 95% CI: −0.26, 0.29; Figure 7A and Table 3). The association between the level of HIV-related stigma and disclosure status is presented in Figure 8B. We observed that not disclosing their HIV status was positively associated with HIV-related stigma (pooled β: 0.75(95%CI: 0.41, 1.09). Furthermore, the level of HIV-related stigma significantly decreased with increasing years of living with HIV, and the pooled estimate was −0.17 (95%CI: −0.31, −0.03) (Figure 8A and Table 2). However, marital status association with HIV-related stigma was not found to be significant (pooled β: 0.01; 95% CI: −0.26, 0.29; Figure 7A and Table 3).

**Table 3:** Risk of bias assessment of 40 studies included in the meta-analysis using the Newcastle–Ottawa Scale.

**References**	**Selection (4)**				**Comparability of Cohorts (2)**		**Outcome (3)**			**Total**
	**Representativeness ofexposed cohort**	**Selection of non-exposed cohort**	**Ascertainment ofexposure**	**Demonstration that the outcome of interest was not present atthe start of study**	**Study control for age and sex**	**Additional factors, controlled for** ≥ **2 variables including comorbidities**	**Assessmentof outcomes**	**Was follow-up long enough for outcomes tooccur**	**Adequacy of follow-up of cohorts**	
Takada et al. (42)	1	1	1	1	1		1	1	1	8
Wedajo (46)	1	1	1	1	1		1	1	1	8
Arinaitwe et al. (32)	1	1	1	1	1		1	1	1	8
Li (36)	1	1	1	1	1		1	1	1	8
Tsai (47)	1	1	1	1	1		1	1	1	8
Nikus Fido et al. (40)	1	1	1	1	1		1	1	1	8
Ncitakalo et al. (48)	1	1	1	1	1		1	1	1	8
Li et al. (49)	1	1	1	1	1		1	1	1	8
Yin et al. (45)	1	1	1	1	1		1	1	1	8
Rayanakorn et al. (19)	1	1	1	1	1		1	1	1	8
Moussa et al. (39)	1	1	1	1	1		1	1	1	8
Xu et al. (44)	1	1	1	1	2		1	1	1	9
Peltzer and Pengpid (20)	1	1	1	1	0		1	1	1	7
Adhikari et al. (30)	1	1	1	1	2		1	1	1	9
Li et al. (50)	1	1	1	1	2		1	1	1	9
Hargreaves et al. (51)	1	1	1	1	1		1	1	1	8
Minja et al. (38)	1	1	1	1	0		1	1	1	7
Antabe et al. (31)	1	1	1	1	1		1	1	1	8
Li et al. (37)	1	1	1	1	2		1	1	1	9
Nyasulu et al. (52)	1	1	1	1	2		1	1	1	9
Feyasa et al. (34)	1	1	1	1	1		1	1	1	8
Tao et al. (53)	1	1	1	1	1		1	1	1	8
Yator et al. (54)	1	1	1	1	1		1	1	1	8
Alemu et al. (16)	1	1	1	1	1		1	1	1	8
Turi et al. (43)	1	1	1	1	2		1	1	1	9
Ajong et al. (55)	1	1	1	1	2		1	1	1	9
Chekole and Tarekegn (56)	1	1	1	1	2		1	1	1	9
Nabunya et al. (57)	1	1	1	1	0		1	1	1	7
Abubakari et al. (58)	1	1	1	1	1		1	1	1	8
Adane et al. (17)	1	1	1	1	2		1	1	1	9
Williams et al. (21)	1	1	1	1	2		1	1	1	9
Algarin et al. (59)	1	1	1	1	1		1	1	1	8
Dahlui et al. (33)	1	1	1	1	2		1	1	1	9
Williams et al. (60)	1	1	1	1	1		1	1	1	8
Deering et al. (61)	1	1	1	1	0		1	1	1	7
Small et al. (62)	1	1	1	1	1		1	1	1	8
Yang et al. (63)	1	1	1	1	1		1	1	1	8
Duko et al. (64)	1	1	1	1	1		1	1	1	8
Spence et al. (41)	1	1	1	1	1		1	1	1	8
Gurmu and Etana (35)	1	1	1	1	1		1	1	1	8

### Quality assessment

The selected studies had a Newcastle–Ottawa score of 7–9, and the studies' quality was considered to be high (Table 3).

### Heterogeneity, sensitivity, and publication bias

**Figure 9:**
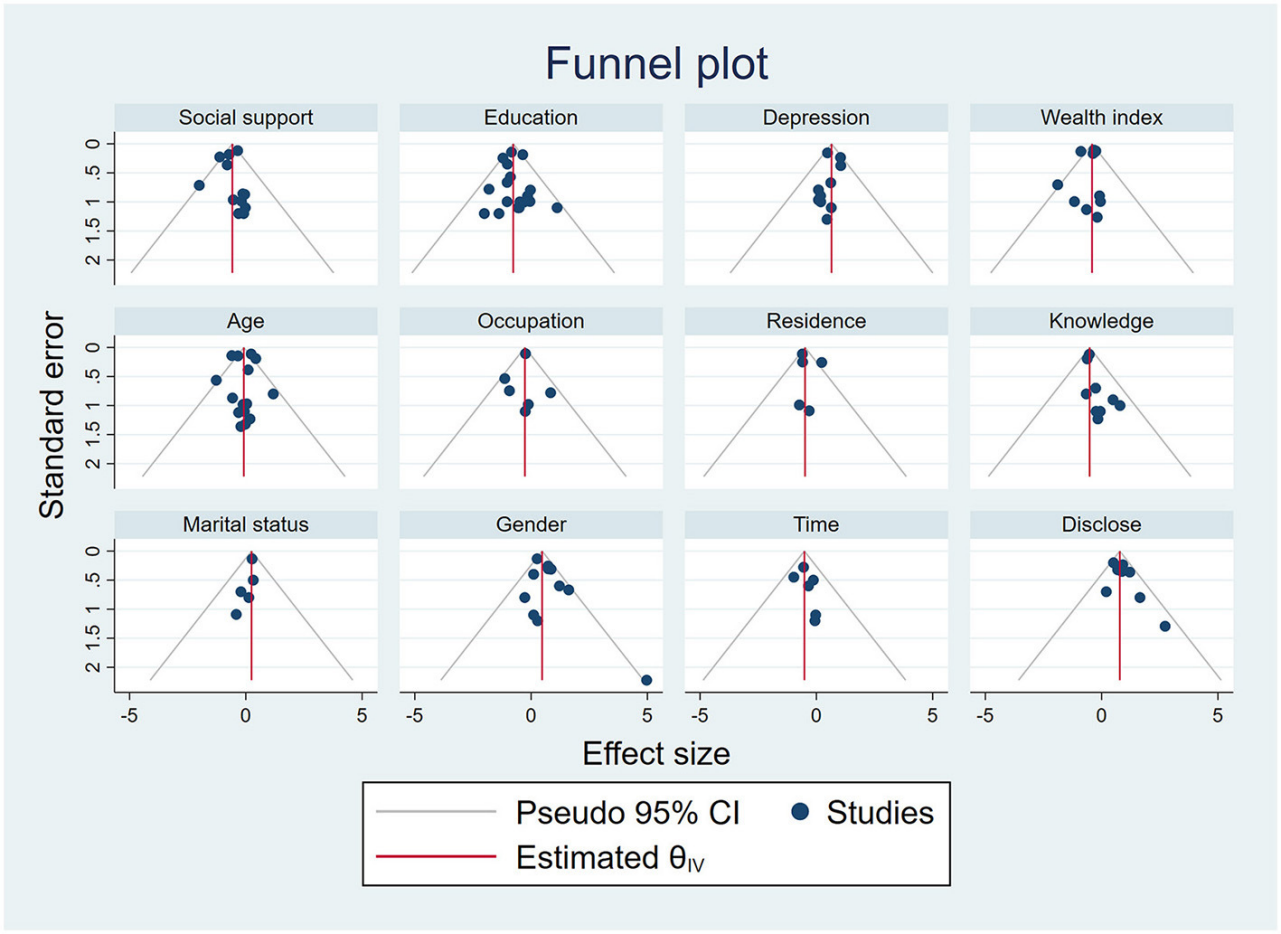
Funnel plots for the publication bias of the effect of age, social support, educational status, depression, employment status, wealth index, gender, residence, knowledge about HIV, marital status, duration since diagnosis, and disclosure status on HIV-related stigma.

The I^2^ values for age, social support, educational status, depression, employment status, wealth index, gender, residence, knowledge about HIV, marital status, duration since diagnosis, and disclosure status demonstrated heterogeneity among the studies under consideration. According to the results of the sensitivity analysis, the estimates for the overall effects of age, social support, educational status, depression, employment status, wealth index, gender, residence, knowledge about HIV, marital status, duration since diagnosis, and disclosure status on the level of HIV-related stigma did not depend on any particular study. According to the funnel plots of the studies included in the meta-analysis, no discernible bias was observed (Figure 9). In addition, neither Egger's regression nor Begg's correlation rank revealed any discernible publication bias (see Table 2).

## Discussion

This review used a series of meta-analyses and data gathered from 40 published studies involving people living with HIV to explore the relationship between a number of health, risk, and sociodemographic factors and HIV-related stigma. In terms of the scale and breadth of risk, health, and sociodemographic factors, our study is by far the largest meta-analysis on the level of HIV-related stigma.

According to our findings, which were consistent with those of earlier research (6, 66), social support significantly reduced the level of HIV-related stigma. According to self-report data from prior studies (67, 68), PLWH with little social support had a higher likelihood of engaging in suicidal behaviors. Social support accessibility provides guidance and relevant information on HIV treatment to people living with HIV (69). Previous reports have suggested that HIV-related stigma could be reduced by improving positive social support, including self-reported social support (70). Thus, social support is crucial for the psychological adjustment of PLWH. Both policymakers and healthcare professionals should look toward enhancing family counseling and support services, such as care for PLWH, and increasing HIV screening among high-risk populations.

In our study, depression was significantly associated with higher levels of HIV-related stigma. This finding was consistent with those of earlier research that found a robust correlation between high levels of HIV-related stigma and high levels of depression (54, 71, 72). It is plausible that stigmatizing beliefs and reactions associated with HIV could evoke feelings of self-blame, guilt, or rejection in people living with HIV (54). Other previous studies (73, 74) on PLWH have discovered that stigma related to HIV is associated with mental health problems, including depression. Thus, to guide future interventions, more research is required to determine how depression affects HIV-related stigma.

Older age at diagnosis was associated with a lower level of HIV-related stigma. Previous studies conducted in different countries also reported similar findings (34, 37, 75). This could be attributed to increased awareness of the disease among older HIV/AIDS patients due to advancement in the education level, exposure to different media, and utilization of the Internet, which promotes HIV-related knowledge. We also observed that women were more likely to face a high level of stigma. This finding was in accordance with those of previous studies (18, 56, 76, 77) that observed that being a woman was significantly associated with a high level of stigma. Existing biases against women may make them more susceptible to HIV-related stigma (40).

We also observed that socioeconomic factors were positively associated with HIV-related stigma. A higher level of education was found to be associated with a lower level of HIV-related stigma in patients. This finding was in accordance with those of previous studies (40, 78) that observed that a lower level of education was significantly associated with a higher level of HIV-related stigma. It has also been demonstrated that an increased level of education reduces HIV-related stigma (48, 79). Moreover, lower household wealth was significantly associated with a high level of HIV-related stigma. This finding was consistent with the findings of previous research that suggested that a higher socioeconomic status helps combat HIV-related stigma (31).

In addition, our data implied that stigmatizing PLWH was associated with psychological issues. For instance, among patients, a lower risk of HIV-related stigma was associated with having high clinical knowledge about HIV and longer years of living with HIV. These findings were consistent with those of previous research (31) that suggested the usefulness of HIV knowledge in rejecting HIV misconceptions and being receptive to PLWH. Therefore, biomedical and clinical knowledge absolve PLWH from being social outcasts who require stigmatization.

### Study limitations

Despite providing pooled estimates from 40 studies across various geographic regions, our analysis has a few limitations. First, some of the included studies had very small sample sizes, which made it difficult to identify any potential influences on high HIV-related stigma. Second, high heterogeneity was observed, which may be due to the wide range of study approaches and sample sizes (ranging from 123 to 56,367 patients).

## Conclusion

Our study indicated a consistent and statistically significant effect of socioeconomic, demographic, psychosocial, and risk variables, including older age, social support, higher educational level, higher socioeconomic status, good knowledge about HIV, disclosure of HIV status, and longer years of living with HIV, which significantly reduced the level of HIV-related stigma. It is crucial to develop complete and comprehensive social strategies through community-level awareness about HIV to tackle systemic HIV-associated stigma.

Moreover, our systematic review results also confirmed that depression, residing in rural areas, female respondents, and poor economic status were significantly associated with a high risk of HIV-related stigma. In addition to advocacy, community economic development or local economic development is essential to build healthy and socially cohesive communities. It would be especially important for policies and programs to effectively integrate agendas across psychosocial, demographic, and socioeconomic interventions to fulfill the UNAIDS 95-95-95 objective by 2030.

## Data availability statement

The original contributions presented in the study are included in the article/supplementary material, further inquiries can be directed to the corresponding author.

## Author contributions

ZD: Conceptualization, Data curation, Formal analysis, Methodology, Software, Visualization, Writing – original draft, Writing – review & editing. TZ: Conceptualization, Methodology, Supervision, Validation, Writing – review & editing.
